# Changes in ideal cardiovascular health among Malawian adults from 2009 to 2017

**DOI:** 10.1038/s41598-022-26340-6

**Published:** 2022-12-19

**Authors:** Calypse Ngwasiri, Sekou Samadoulougou, Kadari Cissé, Leopold Aminde, Fati Kirakoya-Samadoulougou

**Affiliations:** 1 Clinical Research Education Networking and Consultancy (CRENC), RFMR+QFH, Yaoundé, Centre Region Cameroon; 2 Centre de Recherche en Épidémiologie, Biostatistique et Recherche Clinique, Ecole de Santé Publique, Université Libre de Bruxelles, Brussels, Route de Lennik 808, 1070 Belgium; 3 Centre for Research on Planning and Development, Université Laval, Quebec, QC G1V 0A6 Canada; 4 Evaluation Platform on Obesity Prevention, Quebec Heart and Lung Institute, Quebec, QC G1V 4G5 Canada; 5 Departement Biomédical et Santé Publique, Institut de Recherche en Sciences de la Santé, Ouagadougou, Burkina Faso; 6 School of Medicine, Griffith University, Brisbane, Australia

**Keywords:** Cardiovascular diseases, Epidemiology

## Abstract

Ideal Cardiovascular Health (CVH) is a concept defined by the American Heart Association (AHA) as part of its 2020 Impact Goals. Until now, changes in ideal CVH have been poorly evaluated in Sub-Saharan African populations. We aimed to investigate changes in the prevalence of ideal CVH and its components in a population of Malawian adults. Secondary analysis was done on cross-sectional data from 2009 to 2017, obtained from the Malawi STEPS surveys which included 5730 participants aged 25–64 years. CVH metrics categorized into “ideal (6–7 ideal metrics)”, “intermediate (3–5 ideal metrics)” and “poor (0–2 ideal metrics)” were computed using blood pressure, body mass index (BMI), fasting glycaemia, fruit and vegetable intake, physical activity, smoking, and total cholesterol. Sampling weights were used to account for the sampling design, and all estimates were standardised by age and sex using the direct method. The mean participant age across both periods was 40.1 ± 12.4 years. The prevalence of meeting ≥ 6 ideal CVH metrics increased substantially from 9.4% in 2009 to 33.3% in 2017, whereas having ≤ 2 ideal CVH metrics decreased from 7.6% to 0.5% over this time. For the individual metrics, desirable levels of smoking, fruit and vegetable intake, physical activity, blood pressure (BP), total cholesterol and fasting glucose all increased during the study period whilst achievable levels of BMI (< 25 kg/m^2^) declined. From 2009 to 2017, the mean number of ideal CVH metrics was higher in women compared to men (from 2.1% to 5.1% vs 2.0% to 5.0%). However, poor levels of smoking and fruit and vegetable intake were higher in men compared to women (from 27.9% to 23.6% vs. 7.4%% to 1.9% , and from 33.7% to 42.9% vs 30.8% to 34.6%, respectively). Also, whilst achievable levels of BMI rose in men (from 84.4% to 86.2%) the proportion reduced in women (from 72.1% to 67.5% ). Overall, CVH improved in Malawian adults from 2009 to 2017 and was highest in women. However, the prevalence of poor fruit and vegetable intake, and poor smoking remained high in men whilst optimal levels of BMI was declined in women. To improve this situation, individual and population-based strategies that address body mass, smoking and fruit and vegetable intake are warranted for maximal health gains in stemming the development of cardiovascular events.

## Introduction

Cardiovascular disease (CVD) remains the leading cause of premature mortality (deaths before age 70 years) and rising healthcare costs world-wide. The major drivers of CVD include behavioral, cardiometabolic, environmental, and social risk factors, most of which are modifiable^1^. Although the age-standardized mortality rate of CVD is declining in most high-income countries (HICs), similar changes have not been observed in Sub-Saharan Africa (SSA) where people with CVDs are significantly younger compared to their HIC counterparts, posing a threat to socioeconomic development^1–3^. Primary prevention has therefore been increasingly emphasized as a key strategy to reduce mortality and morbidity from CVDs in SSA^2,4^.

The American Heart Association (AHA) introduced the concept of ideal cardiovascular health (CVH) in 2010 in a bid to achieve the goal of reducing deaths from heart disease and stroke by 20% by the year 2020. In their definition, CVH includes a number of metrics consisting four behavioral [smoking, body mass index (BMI), physical activity, and diet] and three metabolic (blood pressure, total cholesterol, and fasting plasma glucose) risk factors for CVD^4^. They defined ideal CVH as the simultaneous presence of both ideal health behaviors (nonsmoking, BMI < 25 kg/m^2^, physical activity at goal levels, and a diet consistent with current guideline recommendations) and ideal health factors (untreated total cholesterol < 200 mg/dL, untreated blood pressure < 120/80 mm Hg, and fasting blood glucose < 100 mg/dL)^4^.

Population-based prospective cohort studies have demonstrated that people with a higher number of ideal CVH metrics have substantially lower risk of CVD^5,6^, CVD mortality^7,8^, cancer mortality^9^ and all-cause mortality^5,10^. Even modest improvements in the CVH status could lead to prolonged years of life free from CVD^6^. These gains have been observed in young adults, middle-aged, and elderly populations^11^. The prevalence and distribution of CVH has been widely studied in different populations. Between 2010 and 2020, pooled estimates show a low global prevalence of ideal CVH, that is, between 3.3% (6–7 ideal CVH metrics) and 16% (5–7 ideal CVH metrics) in the adult population^5,11^. Poor CVH (0–1 ideal metrics) was more common than ideal CVH (6–7 ideal metrics) in most countries, whilst intermediate CVH (2–5 ideal metrics) was the most prevalent^12^. These studies found smoking to be the best CVH metric (71%) and healthy diet the worst (5.8%)^11,12^.

Studies assessing the time trends or changes in CVH levels in the population are scant in SSA. Current evidence shows a declining trend in the proportion of adults meeting all 7 ideal CVH metrics in the USA^7,13,14^, France^15^, and Iran^16^, whilst trends of no improvement over time have also been observed in Canada^17^, Korea^18^ and Ghanaians resident in multiple sites in Europe and Ghana^19^. Published works on the time trends of CVH in important subgroups is also lacking. Although it has been demonstrated that CVH is poorer in men, older persons, and people with a lower socio-economic status^11,15^, little is known on how these relate to changes in CVH over time.

In SSA, the few studies from Ghana^19^, Malawi^20^, South Africa^21^ and Uganda^22^ that assessed the AHA-defined CVH metrics in the adult population focused mainly on the prevalence and distribution of ideal CVH. Another study on a large pan-African population demonstrated CVH index as a surrogate marker for CV risk^23^. However, changes in ideal CVH in the SSA adult population are poorly understood. Such information is necessary to inform policy on which areas individual or population strategies need attention to achieve maximal health gains for primary prevention interventions. Additionally, it could act as a baseline for analyzing the effectiveness of existing or new programs/policies over time. Therefore, the aim of this study was to close this knowledge gap by assessing changes in the CVH metrics in Malawi between 2009 and 2017. We also investigated these changes among the population subgroup (age and sex) as this may provide useful information regarding achievable levels of risk reduction.

## Materials and methods

### Data source

This study included cross-sectional data from the World Health Organization (WHO) STEPwise approach to risk and disease surveillance (WHO STEPS) which is the WHO-recommended framework for collecting, analyzing, and disseminating data on non-communicable diseases (NCDs) and their risk factors^24^. The STEPS is an on-going survey of the health and nutritional status of the non-institutionalized civilian adult population in WHO-member countries. It covers three different levels of “steps” of risk factor assessment involving home interviews (STEPS 1), physical measurements (STEPS 2), and biochemical measurements (STEPS 3).

The focus in each step is obtaining core data on established risk factors that determine the major disease burden. This simple standardized method serves as an entry point for low-income and middle-income countries to get started in NCD prevention and control activities, by building upon each step and adding more complex data depending on the availability of resources.

This study used data from two STEPS surveys in Malawi (2009 and 2017) which both had similar methods of CVH metrics measurement. Changes in ideal CVH was then analyzed from 2009 to 2017. Ethical approval was granted by the Malawi National Research and Ethics Committee. This study was conducted in compliance to the Helsinki Declaration and all participants provided a written informed consent prior to the start of the study.

### Study population and sampling

A multi-stage cluster sampling design was used for each survey to obtain representative data. The sampling process began with the selection of primary sampling units (districts), followed by the selection of enumeration areas within these districts that contain a cluster of households, then the selection of specific households within these segments and finally the selection of individuals within a household. The survey was initially carried out from July 2009 to September 2009 on adults aged 25–64 years and included 5206 participants. A repeat survey in 2017 included 4187 participants aged 18–69 years.

### Data collection and measurements

Socio-demographic (age, sex, level of educational, work status, and place of residence) and behavioral information (tobacco use, healthy diet, and physical activity) were collected in STEP 1. Participant weight, height, and blood pressure measurements were collected in STEP 2. Finally, biochemical measurements to assess blood glucose and cholesterol levels were collected in STEP 3.

- After at least 10 min of rest, the blood pressure of each participant was measured 3 times within a 5-min interval, in a seated position, using an appropriately sized standard cuff sphygmomanometer. The mean value of the second and third reading was considered as the participant’s blood pressure.
- Height was measured using a portable constant tape (Myotape body tape) in standing position with no shoes on, and with a precision of 0.1 cm.
- Weight was measured using a digital scale in light clothing and no shoes on and recorded to the nearest 0.1 kg(kg). BMI was calculated as weight in kg divided by height in squared meters.
- Regarding the biochemical measurements, fasting blood samples were taken according to standard protocols^25^; about 10–12 mls of venous blood was drawn from each participant after at least 8 h of overnight fasting. All samples were stored at − 20 °C and promptly centrifuged (1500 rpm for 10 min at standard room temperature: 21 °C). Fasting plasma glucose was measured via the enzymatic colorimetric method using glucose oxidize, and total cholesterol level was assessed via enzymatic photometric method using cholesterol esterase and cholesterol oxidase.

### Cardiovascular health definitions

The AHA definition was used which includes 3 categories (ideal, intermediate, and poor) for each of the 7 CVH metrics and the overall CVH (Table 1). A participant who took all forms of tobacco (cigarette, pipe, and snuff) daily or occasionally at the time of the interview was categorized as a current smoker. A former smoker referred to anyone with a history of any form of tobacco use or persons who stopped smoking less than 12 months before the time of the interview. The dietary and physical activity components were defined based on the WHO recommendations^26,27^. The definition of ideal CVH excluded individuals who achieved ideal levels of CVH metrics through medication (i.e., antihypertensive, hypoglycemic, or lipid-lowering drugs). The thresholds defining the poor and intermediate categories remained the same for those on such medication(s).

**Table 1:** Definitions of ideal, intermediate, and poor cardiovascular health for each metric.

Metric	AHA definition	Criteria in the present study
Smoking	Ideal: never smoked or have not smoked for more than 12 months	
	Intermediate: stopped smoking for less than 12 months	
	Poor: current smoker	
Fruit and vegetable consumption	Ideal: 4–5 fruit and vegetable components/day	Ideal: consume at least 5 servings of fruit and vegetables per day
	Intermediate: 2–3 fruit and vegetable components/day	Intermediate: 2–4 servings of fruit and vegetables per day
	Poor: 0–1 fruit and vegetable component/day	Poor: 0–1 serving of fruits and vegetables per day
Physical activity	Ideal: ≥ 150 min/week moderate intensity or ≥ 75 min/week vigorous intensity or ≥ 150 min/week moderate + vigorous	Ideal: ≥ 1500 metabolic equivalent of task (MET)-min/week
	Intermediate: 1–149 min/week moderate intensity or 1–74 min/week vigorous intensity or 1–149 min/week moderate + vigorous	Intermediate: 600–1500 MET-min/week
	Poor: no physical activity	Poor: < 600 MET-min/week
Body mass index	Ideal: < 25 kg/m^2^	
	Intermediate: 25–29.9 kg/m^2^	
	Low: ≥ 30 kg/m^2^	
Blood pressure	Ideal: Systolic BP (SBP) < 120 mmHg or diastolic BP (DBP) < 80 mmHg	
	Intermediate: SBP between 120 and 139 mmHg and DBP between 80 and 89 mmHg or subjects on antihypertensive medication	
	Poor: SBP ≥ 140 mmHg or DBP ≥ 90 mmHg	
Total cholesterol	Ideal: < 200 mg/dl	
	Intermediate: 200–239 mg/dl or treatment	
	Poor: ≥ 240 mg/dl	
Glycaemia	Ideal: < 100 mg/dl	
	Intermediate: 100–125 mg/dl or treatment	
	Poor: ≥ 126 mg/dl	
Overall CVH	Poor CVH	0–2 ‘ideal’ metrics
	Intermediate CVH	3–5 ‘ideal’ metrics
	Ideal CVH	6–7 ‘ideal’ metrics

To calculate the total CVH metrics score, each metric was recoded as a binary variable, assigning a score of 1 point to the ‘intermediate’ or ‘ideal’ category and 0 point for the ‘poor’ category. Further, these newly defined variables were summed to obtain a total CVH metric score. This total CVH metric score was then categorized as ‘‘ideal’ (score of 6–7 ideal CVH metrics), ‘intermediate’ (score of 3–5 ideal CVH metrics), and ‘poor’ (score 0–2 ideal CVH metrics)^16^.

### Covariates

The independent variables included for analysis were age, sex, level of education, employment status, and place of residence. Age was categorized as (25–34, 35–44, 45–54, and 55–64), sex (male and female), level of education (none, primary and secondary),employment (unemployed and employed), and residence (urban and rural).

### Data processing and analysis

The distribution of participants’ characteristics was summarised using descriptive statistics. Participants with missing data on all 7 CVH metrics were excluded from the analysis. Sampling weights were used in the analyses to account for the complex nature of the sampling design. Using the direct method, all estimates were standardised by age and sex using the 2018 Malawian population^28^ and the results reported with the 95% confidence intervals. Analysis was performed using STATA software version 17.0. Reporting was done according to the Strengthening Research for Observational Studies (STROBE) guidelines.

## Results

**Figure 1:**
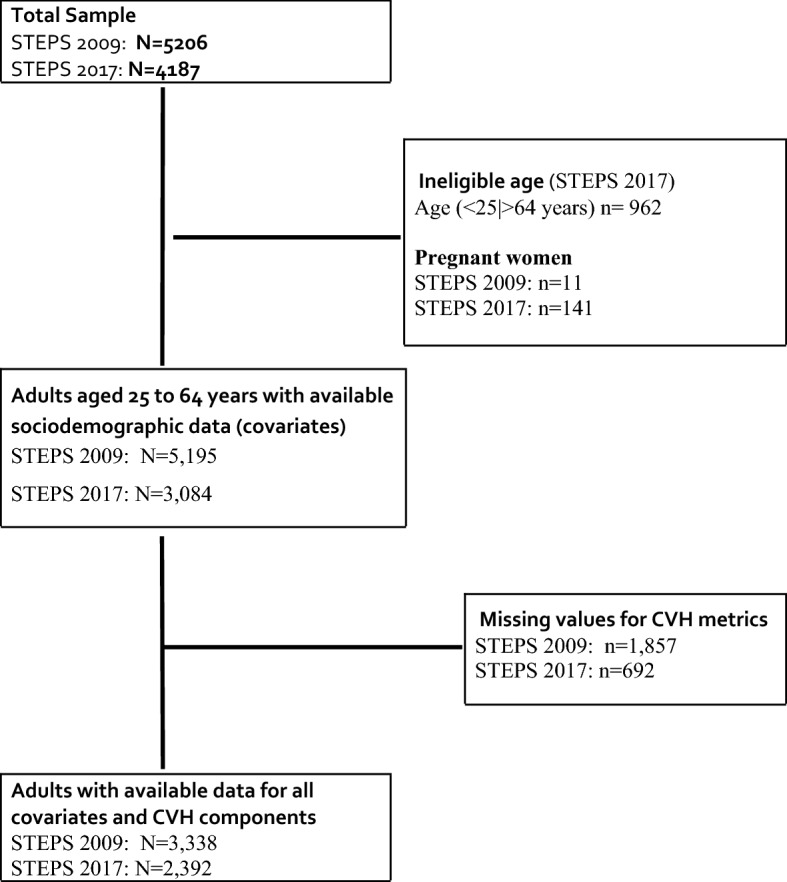
Population flowchart of participants 25–64 years in the 2009 and 2017 STEPS surveys in Malawi.

Overall, 5,195 and 3,084 participants were included in 2009 and 2017, respectively, of whom about three out of four adults fulfilled the eligibility criteria and had no missing value for the CVH metrics. Therefore 3338 and 2392 participants were respectively included in the final analyses across both periods (Fig. 1).

The mean participant age was around 40.1± 12.4 years, more than half of the study population women, and about two-thirds of the participants had some form of primary education. Most individuals were unemployed and largely resided in rural areas (Table 2).

**Table 2:** Sociodemographic characteristics of Malawian adult participants aged 25 to 64 years in 2009 and 2017 (with sampling weights).

	Malawi 2009 (n = 3338)		Malawi 2017 (n = 2392)	
	Frequency	%	Frequency	%
**Age (years)**	39.9 ± 11.4		40.3 ± 10.7	
25–34	1321	39.6	861	40.0
35–44	864	25.9	734	30.7
45–54	646	19.4	481	20.1
55–64	507	15.2	316	13.2
**Gender**				
Female	2293	68.7	1510	63.1
**Level of education**				
None	847	25.4	326	13.6
Primary or less	2050	61.5	1495	62.5
Secondary or more	438	13.1	570	23.8
**Work status**				
Employed	1518	45.5	1014	43.8
Unemployed	1817	54.5	1303	56.2
**Place of residence**				
Urban	358	10.7	460	19.2
Rural	2980	89.3	1932	80.8

### Changes in the prevalence of CVH among the Malawian adult population

**Figure 2:**
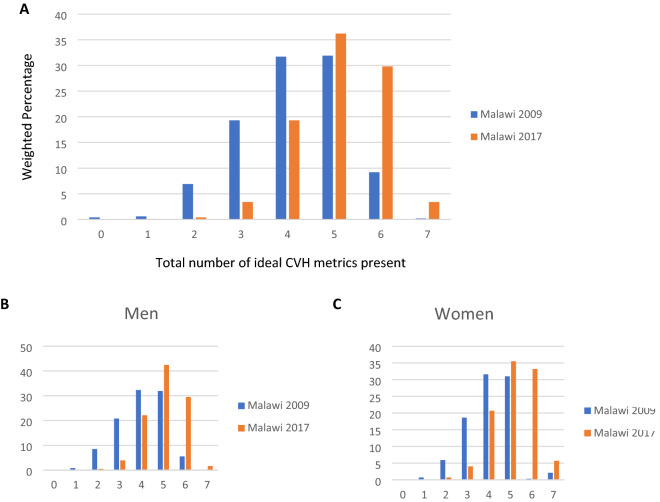
Distribution of the total number of ideal CVH metrics present in Malawi from 2009 to 2017 in the general population (**A**) and according to sex (**B**, **C**).

Among Malawian adults aged 25 years and older, the average number of ideal CVH components increased from 4.2 in 2009 to 5.1 in 2017 after standardizing for age and sex (Table 3). The prevalence of meeting ≥ 6 ideal CVH metrics increased from 9.4% in 2009 to 33.3% in 2017, whereas that of meeting ≤ 2 ideal CVH metrics decreased from 7.6% to 0.5% over this time. As regards the individual metrics, desirable levels of smoking, fruit and vegetable intake, physical activity, blood pressure, total cholesterol and fasting glucose all increased during the study period whilst achievable levels of BMI (< 25 kg/m^2^) declined (Table 3). From 2009 to 2017 in Malawi, women had a higher number ideal CVH metrics compared to men (Fig. 2).

**Table 3:** Age and sex standardized prevalence of CVH metrics in Malawian adults.

Health metrics	STEPS 2009 (n = 3338)	STEPS 2017 (n = 2392)
	Prevalence (95%CI)	Prevalence (95%CI)
**Smoking (any tobacco)**		
Poor	16.4 (15.1–17.8)	11.9 (10.6–13.3)
Intermediate	0.8 (0.4–1.1)	0
Ideal	82.7 (81.3–84.2)	88.1 (86.6–89.4)
**Fruit and vegetable intake**		
Poor	64.6 (62.9–66.4)	38.5 (36.4–40.5)
Intermediate	32.7 (30.9–34.4)	49.0 (46.9–51.1)
Ideal	2.7 (2.1–3.3)	12.5 (11.2–13.9)
**Physical activity**		
Poor	11.1 (10.0–12.2)	5.2 (4.3–6.1)
Intermediate	3.3 (2.7–4.0)	1.8 (1.2–2.3)
Ideal	85.6 (84.4–86.8)	93.0 (92.0–94.0)
**Body mass index (kg/m**^**2**^**)**		
Poor	4.4 (3.8–5.1)	7.1 (6.1–8.0)
Intermediate	17.4 (16.1–18.8)	15.9 (14.4–17.3)
Ideal	78.1 (76.6–79.5)	77.0 (75.3–78.6)
**Blood pressure (mmHg)**		
Poor	32.6 (30.9–34.2)	16.9 (15.4–18.4)
Intermediate	42.3 (40.5–44.1)	36.7 (34.7–38.7)
Ideal	25.1 (23.6–26.6)	46.4 (44.3–48.4)
**Total cholesterol (mg/dl)**		
Poor	32.7 (30.9–34.4)	1.7 (1.2–2.2)
Intermediate	3.4 (2.8–4.1)	5.9 (5.0–6.7)
Ideal	63.8 (62.1–65.6)	92.4 (91.4–93.4)
**Fasting glucose (mg/dl)**		
Poor	15.2 (13.9–16.5)	0.5 (0.2–0.8)
Intermediate	7.4 (6.4–8.4)	0
Ideal	77.4 (75.8–78.9)	99.5 (99.2–99.7)
**CVH metrics scores**		
0–2 CVH metrics	7.6 (6.6–8.6)	0.5 (0.2–0.8)
3–5 CVH metrics	82.9 (81.6–84.3)	58.9 (56.8–60.9)
6–7 CVH metrics	9.4 (8.3–10.4)	33.3 (31.3–35.2)
**Mean CVH metrics (95% CI)**	4.2 (4.1–4.2)	5.1 (5.0–5.1)

#### Changes according to subgroups

Across both periods, disparities were observed between men and women in the prevalence and trend of each CVH metric and overall ideal CVH (Fig. 2 and Table 4). The average number of ideal CVH components increased regardless of sex, however, women had higher levels of ideal CVH scores compared to men in both years (12% vs. 5.5% in 2009, and 38.9% vs. 31% in 2017, respectively). In both groups, a positive change was observed for ideal levels of smoking and physical activity, and was more pronounced in fruit and vegetable intake, total cholesterol, and fasting blood glucose. Whereas achievable levels of blood pressure (< 120/80 mmHg) increased in both groups, the increment was significantly greater in men (from 17.7% to 42.0% vs. 30.5% to 49.5%).

**Table 4:** Age-standardized prevalence of CVH metrics among males and females in Malawi (2009–2017).

Health metrics	Men		Women	
	STEPS 2009 (n = 1045) % (95%CI)	STEPS 2017 (n = 882) % (95%CI)	STEPS 2009 (n = 2293) % (95%CI)	STEPS 2009 (n = 1510) % (95%CI)
**Smoking**				
Poor	27.9 (25.2–30.6)	23.6 (20.8–26.4)	7.4 (6.4–8.5)	1.9 (1.2–2.5)
Intermediate	1.2 (0.5–1.9)	0	0.6 (0.2–0.8)	0
Ideal	70.8 (68.1–73.5)	76.4 (73.6–79.2)	92.0 (90.9–93.0)	98.1 (97.5–98.8)
**Fruit and vegetable**				
Poor	33.7 (30.8–36.5)	42.9 (39.6–46.1)	30.8 (28.9–32.7)	34.6 (32.2–37.0)
Intermediate	64.2 (61.2–67.1)	46.6 (43.3–49.9)	65.8 (63.9–67.7)	51.1 (48.6–53.6)
Ideal	2.1 (1.2–2.9)	10.5 (8.5–12.5)	3.4 (2.6–4.1)	14.2 (12.5–16.0)
**Physical activity**				
Poor	8.7 (7.0–10.4)	4.9 (3.5–6.3)	13.8 (12.4–15.2)	6.1 (4.9–7.3)
Intermediate	2.5 (1.5–3.4)	1.6 (0.8–2.4)	4.2 (3.4–5.0)	1.9 (1.2–2.6)
Ideal	88.8 (86.9–90.7)	93.5 (91.9–95.1)	82.0 (80.4–83.6)	91.9 (90.5–93.2)
**Body mass index**				
Poor	1.8 (1.0–2.6)	2.1 (1.2–3.1)	7.2 (6.1–8.2)	12.0 (10.3–13.6)
Intermediate	13.8 (11.7–15.9)	11.7 (9.6–13.8)	20.7 (19.0–22.3)	20.5 (18.5–22.6)
Ideal	84.4 (82.2–86.6)	86.2 (83.9–88.4)	72.1 (70.3–74.0)	67.5 (65.1–69.8)
**Blood pressure**				
Poor	38.5 (35.6–41.4)	16.9 (14.4–19.3)	30.5 (28.8–32.3)	18.9(17.0–20.8)
Intermediate	43.8 (40.8–46.8)	41.1 (37.9–44.3)	38.9 (36.9–40.9)	31.6 (29.2–33.9)
Ideal	17.7 (15.4–20.0)	42.0 (38.7–45.2)	30.5 (28.7–32.3)	49.5 (47.1–52.0)
**Total cholesterol**				
Poor	35.9 (30.0–35.8)	1.8 (0.9–2.6)	32.3(30.4–34.3)	1.8 (1.2–2.5)
Intermediate	3.2 (2.1–4.2)	4.5 (3.2–5.9)	4.2(3.4–5.0)	8.1(6.8–9.5)
Ideal	63.9 (61.0–66.8)	93.6 (92.1–95.2)	63.4(61.4–65.3)	90.0(88.5–91.5)
**Fasting glucose**				
Poor	15.5 (13.3–17.7)	0.7 (0.1–1.2)	15.2(13.7–16.7)	0.6 (0.2–1.0)
Intermediate	9.4 (7.6–11.1)	0	5.8(4.9–6.8)	0
Ideal	75.1 (72.5–77.7)	99.3 (98.8–99.8)	78.9(77.3–80.6)	99.4 (99.0–99.8)
**Scores**				
0–2 CVH metrics	9.2 (7.5–11.0)	0.5 (0.0–0.9)	6.6 (5.7–7.8)	0.8 (0.3–1.3)
3–5 CVH metrics	85.2 (83.0–87.3)	68.5 (65.4–71.6)	81.2 (79.6–82.8)	60.2 (57.7–62.8)
6–7 CVH metrics	5.5 (4.1–6.9)	31.0 (27.9–34.1)	12.0(10.7–13.4)	38.9 (36.4–41.5)
**Average score**	2.0 (1.9–2.1)	5.0 (4.9–5.1)	2.1 (2.0–2.1)	5.1 (5.0–5.2)

Although ideal levels of smoking and fruit and vegetable consumption increased in both sexes across the study period, the overall prevalence was low. Poor smoking was higher in men compared to women from 2009 to 2017 (27.9% to 23.6% vs 7.4% to 1.9%), whilst the prevalence of poor fruit and vegetable intake was high, but similar in bioth sex groups (from 33.7% to 42.9% vs 30.8% to 34.6%) Also, whilst optimal levels of BMI (< 25 kg/m^2^) surged in men, the proportion declined in women (from 72.1% in 2009 to 67.5% in 2017) (Table 4).

## Discussion

The present study found that overall CVH improved in Malawian adults from 2009 until 2017. This improvement was present across both sexes, but highest in the women. Furthermore, optimal levels of BMI declined overall especially in women, whilst positive changes were observed for ideal levels of fruit and vegetable intake, total cholesterol, and fasting blood glucose regardless of age or sex. However, the prevalence of poor levels of smoking remained high in men whereas undesireable levels of fruit and vegetable consumption were high in both men and women.

Differences in the composition of cohorts, the data collection periods, and different definitions used for CVH makes comparison with other studies on time trends in CVH difficult. Regardless, our study is among the first to report an overall improvement in CVH in a community-based population in SSA. The overall advancements in ideal CVH between 2009 and 2017 was due to improvements in all metrics but body mass. Our findings are consistent with reports from High Income Countries (HICs) which show significant improvement in CVH. In the USA, significant improvement in CVH was reported among healthcare employees (from 0.3% to 0.6%) within a 3-year period (2011–14)^29^. Similarly, in France^15^ and Denmark^30^, substantial improvements in CVH (6.7–15.0% and 1.6–9%) was seen in a community-based sample of adults over a 9 years (1992–2011) and 28 years (1978–2006), respectively. In contrast to these and our findings, studies from Canada^17^, South Korea^18^ and Ghana^19^ have revealed trends of no improvement over time. We also found that CVH was higher in women at any point in time which was in accordance with previous studies^7,31^.

The overall improvement of CVH might arguably be thanks to the success of public health efforts in implementing primary prevention interventions which have been shown to be cost-effective in CVD prevention especially in SSA^32^. In Malawi, a National Non-Communicable Disease (NCD) Action Plan was developed and included in the health sector strategic plan from 2012 to 2016. Informed by the Malawian NCD poverty commission, this action plan focused mainly on the poorest population groups, and targeted both primary and secondary prevention interventions against harmful alcohol use, smoking, unhealthy diet and physical inactivity^33^. Similar to other SSA countries, emphasis was done on individual-level interventions targeting high blood pressure and high cholesterol, and on population-based interventions targeting smoking cessation^32^. There was less emphasis on interventions that addressed physical inactivity, low fruit and vegetable consumption and body mass, which is mirrored in our findings, where higher levels of low fruit and vegetable consumption (poor fruit and vegetable) and obesity (poor BMI) remained persistent despite an overall CVH improvement. This trend was also observed in other population-based studies^7,14,17,30,34^. If allowed to continue, the increasing prevalence of obesity (which has a downstream impact on other CVD risk factors such as blood pressure and blood glucose) could slow improvements in CVH via offsetting the positive effects from the decline in other CVD risk factors as observed in France^15^.

Smoking and physical activity were the best metrics in our study with the highest ideal levels regardless of sex, whilst fruit and vegetable consumption the worst, with highest poor levels. This emphasizes the continuing importance of primary prevention to preserve and sustain ideal CVH (like smoking and physical activity), or to restore Ideal levels (fruit and vegetable intake). This could be achieved via population-level interventions, especially legislative interventions which have been shown to offer good value for money in SSA^32,35,36^.

Despite less emphasis on physical inactivity, we found high ideal levels in this individual CVH metrics. The high level of physical activity may be due to a high proportion of farming among the participants, as the majority were resident in rural areas, with about 80% of its population heavily reliant on agriculture for subsistence^37^.

Taken together, the current study illustrates changes in CVH in Malawi over 8 years, and possible underlying components responsible for such changes. However, our study had some limitations that warrant consideration. The cross-sectional design and assessment of some variables by self-reporting (physical activity, smoking and diet metrics) may have led to misclassification errors. Such misclassification is likely to have overestimated the proportion of adults in the ideal category owing to social desirability bias. Also, smoking might have been under-reported due to the tendency to provide responses that are socially desirable. Finally, the high proportion of missing values for each CVH metric may have biased our results. Despite these limitations, our study provides important data on the changes in ideal CVH in a SSA population. It uses a large nationally representative sample with standardized methods and measures, and may serve as evidence for sex- and age-specific initiatives to sustain and further improve ideal CVH in Malawi and other SSA populations.

## Conclusion

Overall, CVH improved in Malawi over 8 years, but the average proportion of ideal CVH metrics remains low. This positive change in CVH was more prominent in smoking, physical activity, total cholesterol and fasting glucose. Other components of ideal CVH also had a positive change but ideal levels of BMI declined. Furthermore, the improvement in CVH was weaker in men for whom poor levels of smoking remained very high. Poor levels of fruit and vegetable consumption also remaind high despite a positive change in the ideal category over the study period. Therefore, to obtain maximal health gains and stem the CVD epidemic, both individual and population-based strategies are required with focus on body mass, low fruit and vegetable consumption, and smoking. There is need for increased multi-faceted health promotion and prevention strategies targeting key determinants of poor CVH.

## Supplementary Information

Supplementary Information 1.

Supplementary Information 2.

Supplementary Information 3.

Supplementary Information 4.

Supplementary Information 5.

Supplementary Information 6.

## Data Availability

The datasets generated and used for analyses were provided by the WHO NCD Surveillance Monitoring and Reporting Unit. These datasets (mwi2009.dta, mwi 2017.dta and Agsexstrut1.dta) and the do.files of each analysis report are available within the article (and its supplementary information files).
